# Experimental Investigation on Mechanical and Acoustic Emission Characteristics of Gassy Coal under Different Stress Paths

**DOI:** 10.3390/ijerph19137873

**Published:** 2022-06-27

**Authors:** Jie Liu, Qiuping Li, Jinduo Li, Zaiquan Wang, Shouqing Lu

**Affiliations:** 1 Key Lab of Industrial Fluid Energy Conservation and Pollution Control, Qingdao University of Technology, Ministry of Education, Qingdao 266520, China; tqlfighting@qut.edu.cn (Q.L.); lushouqing@qut.edu.cn (S.L.); 2 Shandong Key Industry Field Accident Prevention Technology Research Center (Non-Ferrous Metallurgy), Qingdao 266520, China; 3 TSMC Nanjing Company Limited, Nanjing 211800, China; jdlio@tsmc.com; 4 School of Civil Engineering, Qingdao University of Technology, Qingdao 266520, China; wangchunyuan@qut.edu.cn

**Keywords:** gas-bearing coal, fixed axial stress and unloading confining stress, damage coefficient, acoustic emission

## Abstract

Coal mining leads to stress loading–unloading variation in front of the working face, which influences the occurrence of disasters. In order to study the influence mechanism of stress loading–unloading to the coal failure, a series of experiments of gas-bearing coal deformation and failure under triaxial stress were conducted and acoustic emission (AE) was monitored. In this study, the effect of gas pressure on the mechanical behavior of gas-bearing coal in conventional triaxial stress (CTS) experiments and fixed axial stress and unloading confining stress (FASUCS) experiments was analyzed, and the damage evolution rules of gas-bearing coal in the CTS experiments and FASUCS experiments were determined using AE. The results show that with the increasing of gas pressure, the peak strength and peak strain of gas-bearing coal in the CTS experiments and FASUCS experiments gradually decrease, and the peak of AE ring-down counts lags behind the peak strength. Compared with the CTS experiments, the strength of gas-bearing coal in the FASUCS experiments is lower and the precursor information appears later. The trends in calculated stress and damage coefficient D are consistent with the stress path during unloading, and both begin to rise sharply after the sample enters the plastic stage. Therefore, AE ring-down counts, damage coefficient D, and calculated stress can be used as precursor information for failure of coal and rock, which has great significance for the further study of coal-rock material and for early hazard warning.

## 1. Introduction

Gas-bearing coal is a gas–solid multiphase discontinuous medium containing complex ir-regular primary cracks and secondary fractures [1,2,3]. During coal mining, changes in the external stress field cause the migration and flow of gas inside the coal body, affecting the adsorption and desorption of gas [4,5,6]. At the same time, the adsorption and desorption of gas will also lead to the change in the mechanical properties of the coal body [7]. The stress state of the original rock where the coal seam is located is often altered [8,9], and coal-rock will be intermittently unloaded [10,11]. At this time, the coal-rock is more prone to instability and damage, which may lead to coal and gas outburst accidents or other hazards [12,13].

Previous scholars have studied the mechanical properties of coal and rock under true triaxial [14] and unloading conditions [15]. Palm studied the stress–strain relations for uniform monotonic deformation under triaxial loading [16]. Taheri et al. studied the strength and deformation characteristics of cement-mixed gravelly soil in multiple-step triaxial compression [17]. Thomas et al. carried out an experimental study on the damage evolution of brittle rock under triaxial confinement with full circumferential strain control [18]. The results showed that there is a difference in the ultimate strength of coal during the processes of loading and unloading, and the shear failure and tensile–shear composite failure modes occur in coal under different loading and unloading paths. However, coal under natural conditions is mostly in the form of gas-bearing coal. Chen et al. described the evolution of damage during cyclic loading and unloading under various confining pressure, which was based on the theory of elastic–plastic damage mechanics [19]. Pirzada et al. studied CO_2_-sorption-induced damage in coals in unconfined and confined stress states [20]. They showed that the existence of gas has a key influence on the strength and deformation characteristics of coal-rock. Some scholars studied gas-bearing coal in the triaxial state more deeply. Wang et al. carried out conventional triaxial creep tests of gas-bearing coal with different load levels and conducted a mathematical fitting study on the experimental curves [21]. Xu et al. analyzed the effects of gas pressure on surface modulus, strength, and failure time using unloading tests under different gas pressure conditions [22].

Acoustic emission (AE) is an elastic wave in a solid caused by an ir-reversible change in the internal structure of the material [23,24]. In the 1930s, Obert discovered AE phenomena and applied AE techniques to monitor ore body stability and predict rock burst [25]. Dunegan et al. adjusted the range of AE test frequencies to the ultrasonic range (tens to hundreds of kHz), which greatly reduced the influence of background noise and laid a good foundation for the practical application of AE [26]. Since then, AE technology has been applied to geotechnical and concrete engineering around the world [27,28,29], especially to monitor and predict rock disasters and coal and gas outbursts in underground mines [30,31]. Kurita carried out some research on AE of rock expansion before rock failure [32]. Shkuratnik et al. studied the AE response in coal samples under uniaxial and triaxial compression and analyzed AE counts, cumulative counts, and stress and strain relationships [33]. Kong et al. studied the acoustic emission law during gas coal loading and analyzed the damage evolution [34]. Su et al. studied the difference of AE characteristics in coal fractures with different stress paths under pseudo-triaxial conditions [35]. Browning et al. discussed the AE characteristics of sandstone under true triaxial loading and carried out a typing study of the effect of crack angle on acoustic emission signals [36]. The research shows that the AE count and the cumulative count, respectively, characterize the processes of coal failure from different angles and the brittle fracture of the tensile–shear composite in the unloaded state. In recent years, AE has been widely used in coal mine monitoring and early warning systems, especially for hazard warnings for gas-bearing coal.

Previous scholars have begun to simulate the mechanical state under real mining stress conditions through more complicated experimental designs. However, most of them are pseudo-triaxial tests in which confining stress cannot be controlled in different directions. A few studies that have implemented true triaxial tests are often directed at rock or non-gas-bearing coal. A true triaxial test of gas-bearing coal is lacking. In view of these deficiencies, this study conducted a true triaxial test of gas-bearing coal while recording the AE signal. The damage mode and precursor signals of gas-bearing coal in mining stress conditions were studied from the following aspects: mechanical characteristics, AE characteristics, and damage mechanism. This test is of practical significance for studying the deformation mechanism and obtaining precursor warning information for gas and coal-rock hazards.

## 2. Materials and Methods

### 2.1. Experimental System

**Figure 1:**
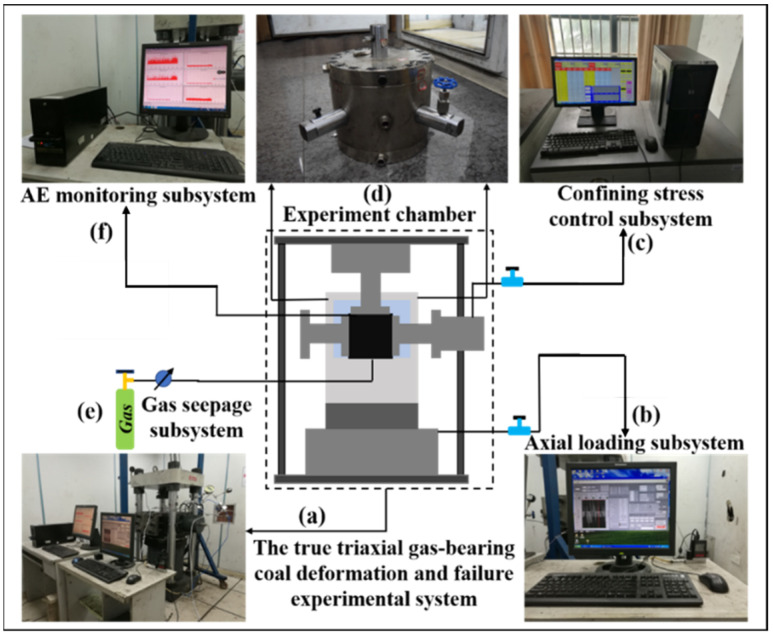
Schematic diagram of the experimental system. (**a**) The true triaxial gas-bearing coal deformation and failure experimental system; (**b**) axial loading subsystem; (**c**) confining stress control subsystem; (**d**) experiment chamber; (**e**) gas seepage subsystem; (**f**) AE monitoring subsystem.

The true triaxial deformation and failure test of gas-bearing coal included an axial loading and unloading system, a lateral pressure loading and unloading system, a sealed cavity, an AE monitoring system, and a gas seepage system. The schematic is shown in Figure 1. The experimental system can simultaneously evaluate mechanical deformation of multiphase (gas, solid, liquid) porous media, adsorption and expansion deformation, failure, seepage, coupled loading and unloading gas–liquid–solid seepage, and gas/hydraulic fracture characteristics. It can measure the dynamic change of permeability in three dimensions: X, Y, and Z; and it can synchronously monitor the AE responses during the experiment. It can simulate the loading and unloading process caused by deep rock stress and mining stress in natural environments. The axial compression loading subsystem and the lateral loading subsystem control the loading and unloading stress of the coal-rock sample in the central sealed cavity in all three dimensions. At the same time, the crack propagation and damage of the multiphase medium under load are monitored.

#### 2.1.1. Triaxial Loading and Unloading System

The triaxial cylinder was installed in the servo-controlled hydraulic machine, as shown in Figure 1. The YAW4306 mechanical test equipment included presses, numerical control systems, and Power Test V3.3 control programs. The loading system provided closed loop control, constant stress control, and stress retention. The maximum load capacity was 3000 kN, and the loading speed could be adjusted between 60 and 60,000 N/s with an accuracy of ±1%. The loading device had two control modes: displacement control and force control. These modes are available for uniaxial compression and stretching, cyclic loading, creep testing, and other tests. The main stress ($\sigma_{1}$) was applied by the servo-controlled hydraulic cylinder. The intermediate principal stress ($\sigma_{2}$) and the secondary principal stress ($\sigma_{3}$) were supplied by three external hydraulic pumps.

#### 2.1.2. AE Monitoring System

The Express-8 AE system (Physical Acoustics Corporation, Princeton, NJ, USA) was used for AE monitoring. The system has 24 AE data-acquisition channels. R15α sensor manufactured by Physical Acoustics Corporation was used in the experiments. The signal collected by the AE sensor is amplified by a preamplifier and transmitted to the 16-bit analog-to-digital (A/D) converter module. The parameters and data in the digital signal were stored in a buffer, and then transferred to a computer for further processing and display.

### 2.2. Sample Preparation

The samples were taken from Henan Province, China. The samples were processed according to the standards specified in the international rock mechanical test. The sample is a cube of size 100 mm × 100 mm × 100 mm. The roughness of each surface did not exceed ±0.5 mm to ensure that the sample was evenly stressed during compression and to avoid stress concentration that could affect the accuracy of the experimental results. In total, 21 samples were required for this test, which were divided into 7 groups. The first 4 groups were used for the conventional triaxial test, and the last 3 groups were used for the unloading test.

### 2.3. Experimental Program

**Figure 2:**
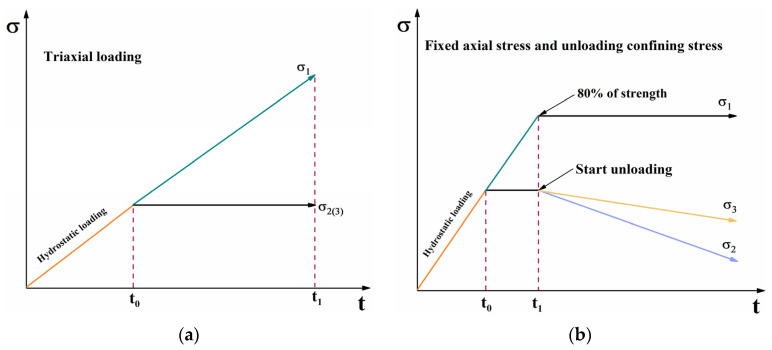
Stress path map. (**a**) Triaxial loading. (**b**) Fixed axial stress and unloading confining stress.

In order to simulate the mining process in the working face, two test paths, namely, CTS—Figure 2a—experiments and FASUCS—Figure 2b—experiments, were selected. We selected twelve samples to determine the conventional triaxial compressive strength of coal when it adsorbed gas at different pressures of 0 MPa, 0.2 MPa, 0.5 MPa, 0.8 MPa, respectively. Nine samples were selected to conduct the experiments of fixed axial stress and unloading confining stress. The test conditions of each coal sample are shown in Table 1. The specific experiment program is as follows:

1. The stress of 1 MPa was applied to the chamber at the axial direction. Then, the coal and chamber were vacuumed for 8 h, and gas at the pressure of 0.2 MPa, 0.5 MPa, and 0.8 MPa was injected into the chamber and absorbed for 24 h.
2. Before the test, the AE sensors were placed on both sides of the axial rigid pressure bar and fixed with magnetic suction. The sensor and the pressure bar were connected via Vaseline. After that, pencil lead break was made to ensure that the AE signal could be adequately collected. The preamplifier threshold was set to 45 dB. A slight tap before the experiment was performed to determine that the two channels could acquire the AE signal well.
3. The conventional triaxial test applied stress to $\sigma_{1}$ = $\sigma_{2}$ = $\sigma_{3}$ = 19 MPa using the hydrostatic loading method, and the axial pressure was increased to the sample failure at a loading speed of 500 N/s. The strength of the coal sample was obtained.
4. In the experiments, the stress of $\sigma_{1}$, $\sigma_{2}$, and $\sigma_{3}$ were synchronously applied to 19 MPa, then the axial stress was increased to 80–90% of the conventional triaxial strength. After that, $\sigma_{2}$ and $\sigma_{3}$ were simultaneously unloaded at 0.01 MPa/s and 0.005 MPa/s, respectively, until the sample failure. AE data acquisition took place during the entire loading process of the conventional triaxial text. For the unloading test, AE data acquisition was started after $\sigma_{1}$ = 12 MPa. All data were collected until the sample failed.

## 3. Results

### 3.1. Conventional Triaxial Test

#### 3.1.1. Mechanical Properties and Deformation Characteristics

**Figure 3:**
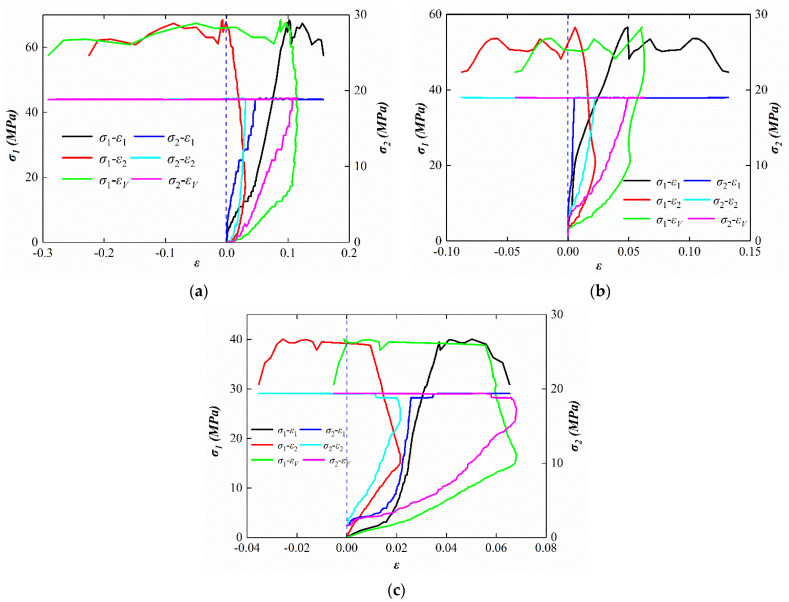
Conventional triaxial test stress–strain curves at different gas pressure. (**a**) Stress–strain curve of 0.2 MPa. (**b**) Stress–strain curve of 0.5 MPa. (**c**) Stress–strain curve of 0.8 MPa.

Three parallel tests were set up for each experiment, and the triaxial intensity values were averaged from three trials. Each of the following sets of tests only shows the curve from one of the trials. Figure 3 shows conventional triaxial test stress–strain curves (samples 5, 7, 11). When the gas pressure increased, the peak stress of the coal sample decreased under the conventional triaxial stress. When the strain applied to the load was reduced, the time required for failure was shorter. The sample did not expand under the condition of no gas pressure. In the presence of gas pressure, the sample showed a certain degree of expansion. The peak strength of gas-bearing coal in the CTS experiments were 68.46 MPa, 55.93 MPa, and 40.04 MPa at the gas pressure of 0.2 MPa, 0.5 MPa, and 0.8 MPa, respectively, which reveals that gas influenced the peak strength of gas-bearing coal in the CTS experiments.

#### 3.1.2. Acoustic Emission Characteristics

**Figure 4:**
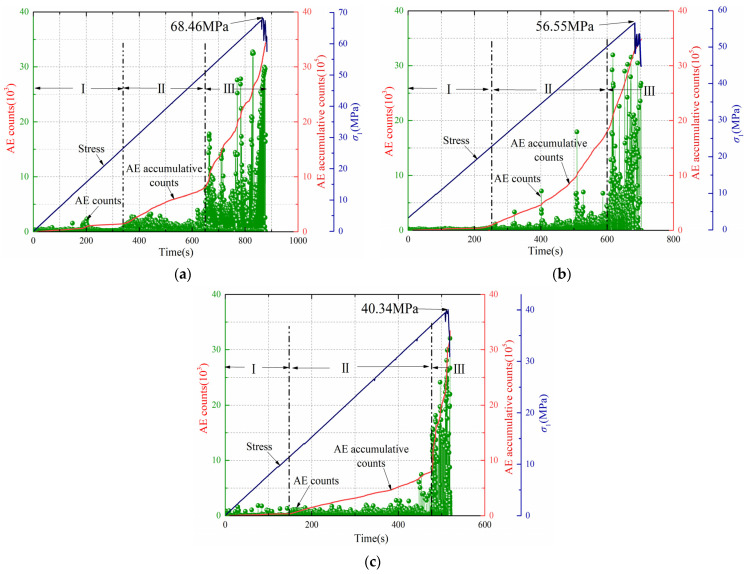
Acoustic emission response of the conventional triaxial test. (**a**) Samples 5. (**b**) Samples 7. (**c**) Samples 11.

Figure 4 shows the AE diagram for the conventional triaxial test of gas-bearing coal (samples 5, 7, 11). The AE signals can be divided into three stages: (I) quiet period, (II) active period, and (III) acceleration period. The compact and elasticity stages of coal occur in the quiet period. At this stage, closure and elastic deformation of the pore fractures occur. Therefore, only a few AE signals are generated, and the cumulative ring-down count is also stable.

The active period refers to the later period of time after the coal enters the plastic stage. The number of AE ring-down counts during this period increases compared with the quiet period. At 0.5 MPa shown in Figure 4b, there were three abrupt increases in AE ring-down counts, but the durations were very short. At 0.2 MPa, shown in Figure 4a, and 0.8 MPa, shown in Figure 4c, AE ring-down counts increased steadily. During this period, the slope of the cumulative ring-down counting curve increased, but the overall trend of increase was gentle. At this time, the coal sample entered the stage of plastic deformation, and the pore fissures began to develop.

The acceleration period refers to the period from macrofracture development to failure (stress drop) of the coal. Acoustic emission ring-down counts increased sharply during this period. The slope of the cumulative curve of AE ring-down counts rises sharply until the stress reaches the peak and the coal body breaks. Therefore, the AE signals during the acceleration period can be regarded as precursors to coal damage. At 0.2 MPa, 0.5 MPa, and 0.8 MPa, the time from the beginning of the acceleration period to the time of coal damage was 232 s, 108 s, and 42 s, respectively. The higher the gas pressure is, the longer the duration of the quiet period and the active period is and the shorter the duration of the acceleration period is, which indicates that with the increasing of gas pressure, the speed from crack expansion to complete failure of gas-bearing coal is accelerated.

### 3.2. Fixed Axial Stress and Unloading Confining Stress

#### 3.2.1. Mechanical Properties and Deformation Characteristics

**Figure 5:**
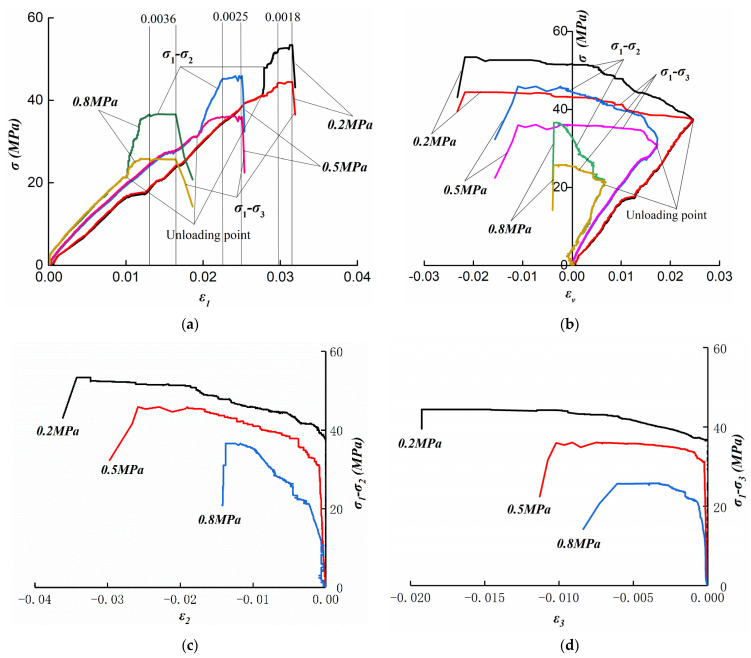
Stress–strain curves for the unloading test. (**a**) *ε*_1_−(*σ*_1_−*σ*_2_) and (*σ*_1_−*σ*_3_) curves; (**b**) ε_v_−(*σ*_1_−*σ*_2_) and (*σ*_1_−*σ*_3_) curves; (**c**) ε_2_−(*σ*_1_−*σ*_2_) curves; (**d**) ε_3_−(*σ*_1_−*σ*_3_) curves.

According to the peak stress values at different gas pressures obtained from the conventional triaxial stress experiments, the fixed axial pressure is 58 MPa when gas pressure is 0.2 MPa, 50 MPa when gas pressure is 0.5 MPa, and 40 MPa when gas pressure is 0.8 MPa. Figure 5 shows the stress–strain curve of specimens 13, 18, and 19. In the experiments, the difference in stress was used to measure the strength of sample, namely, $\sigma_{1}$-$\sigma_{2}$ is the stress difference in direction A and $\sigma_{1}$-$\sigma_{3}$ is the stress difference in direction B.

When gas pressure increased, the radial strain, the axial strain, the volume strain, and the stress difference from unloading to cracking all decreased. Figure 5a shows that when the lateral stress unloads, the stress–strain curve gradually increases, and the slope increases significantly before the unloading. When the gas pressure is 0.2 MPa, the stress difference ($\sigma_{1}$-$\sigma_{2}$ and $\sigma_{1}$-$\sigma_{3}$) of the coal sample is 53.42 MPa and 44.43 MPa; when the gas pressure is 0.5 MPa, the stress difference of the coal sample is 45.93 MPa and 36.13 MPa; when the gas pressure is 0.8 MPa, the maximum stress difference of the coal sample is 36.66 MPa and 25.83 MPa. It obviously indicates that the strength of coal decreases with the increasing of gas pressure. In the experiments, before unloading the confining pressure, the stress–strain curve increases linearly, the specimen is in elastic deformation, and the elastic modulus remains constant. When the confining stress begins to unload, the slope of the stress−strain curve increases rapidly, and the difference between $\sigma_{1}$-$\sigma_{2}$ and $\sigma_{1}$-$\sigma_{3}$ increases gradually. The strain increases rapidly before the complete fracture. However, the increment of strain rises with the increase in gas pressure. With the increase in gas pressure from 0.2 MPa to 0.8 MPa, the strain increment rises from 0.0018 to 0.0036.

Figure 5b shows that before confining-stress unloading, the volumetric strain increases continuously, and the sample is compressed. After confining pressure begins to unload, the volumetric strain decreases continuously. Compared with the axial stress difference strain curve, it can be found that at the beginning of confining-pressure unloading, the axial strain changes slightly. At this time, the reason for the decrease in volumetric strain is that the horizontal strain increases rapidly, which leads to the expansion deformation of the sample. With the increasing of gas pressure, the expansion deformation decreases gradually. Figure 5c,d show that the radial strain begins to increase gradually after the lateral pressure begins to be removed. Moreover, because of the different speeds of lateral-pressure relief in the two directions, the growth rate and absolute value of *ε*_2_ during rupture are significantly higher than *ε*_3_, and the *ε*_3_ curve is more stable.

**Figure 6:**
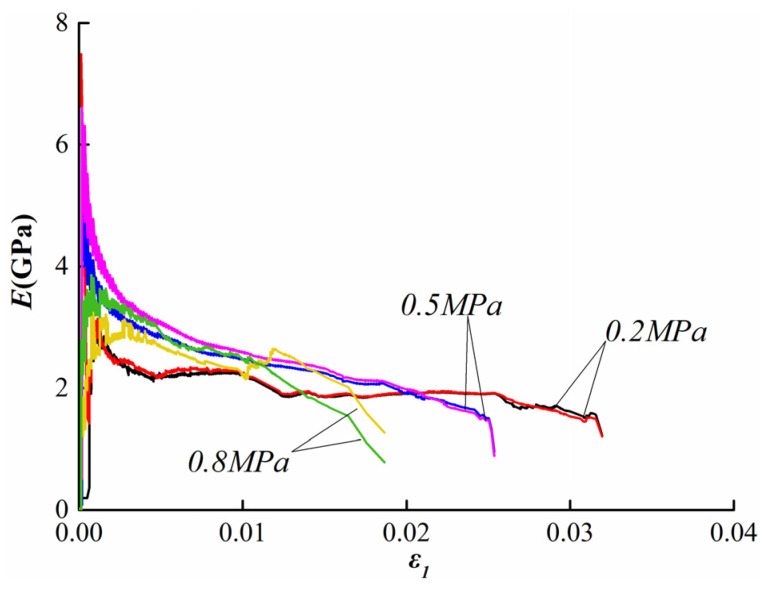
Deformation modulus curve.

**Figure 7:**
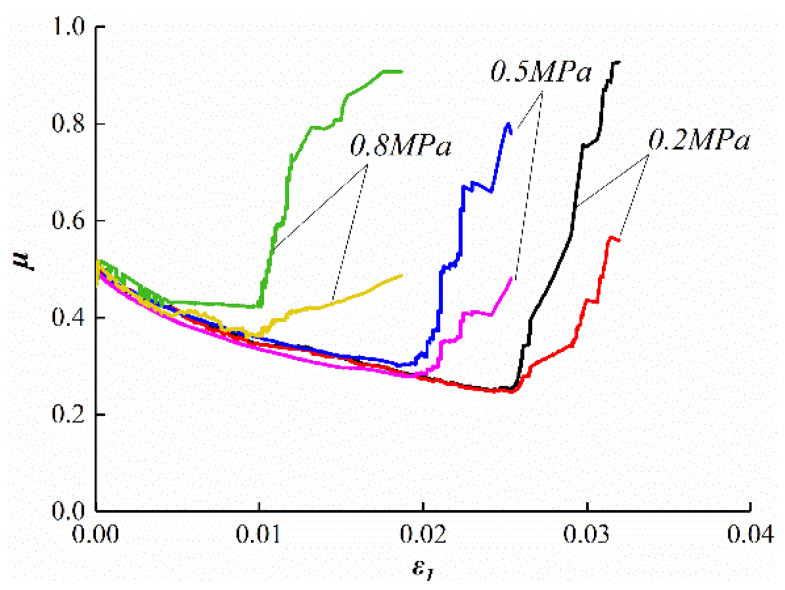
Poisson’s ratio curve.

Axial strain and radial strain under different experimental conditions show different rules; therefore, the deformation modulus and Poisson’s ratio of coal body will change. The deformation modulus and Poisson’s ratio can be calculated by Formula (1) [37].

(1) $$\begin{array}{l} E=(\sigma_{1}-2\mu \sigma_{2})/\varepsilon_{1}\\ \mu =(B\sigma_{1}-\sigma_{2})/[\sigma_{2}(2B-1)-\sigma_{1}]\\ B=\varepsilon_{2}/\varepsilon_{1} \end{array}$$

where *E* is the deformation modulus and *μ* is the Poisson’s ratio. Figure 6 shows the deformation modulus curve of the coal in the FASUCS experiments at different gas pressure conditions. Before the failure of coal samples, the deformation modulus of samples under gas pressures of 0.5 MPa and 0.8 Mpa is larger than that under gas pressure of 0.2 Mpa. Figure 7 shows the Poisson’s ratio curve of the coal in the FASUCS experiments at different gas pressure conditions. Under different gas pressures, the curve of the elastic stage shows a downward trend, and Poisson’s ratio decreases gradually. When the confining stress unloaded, the Poisson’s ratio increases. A comparison of the three tests shows that with the increasing of gas pressure, the Poisson’s ratio is decreasing and the curve is steepening.

#### 3.2.2. Acoustic Emission Characteristics

**Figure 8:**
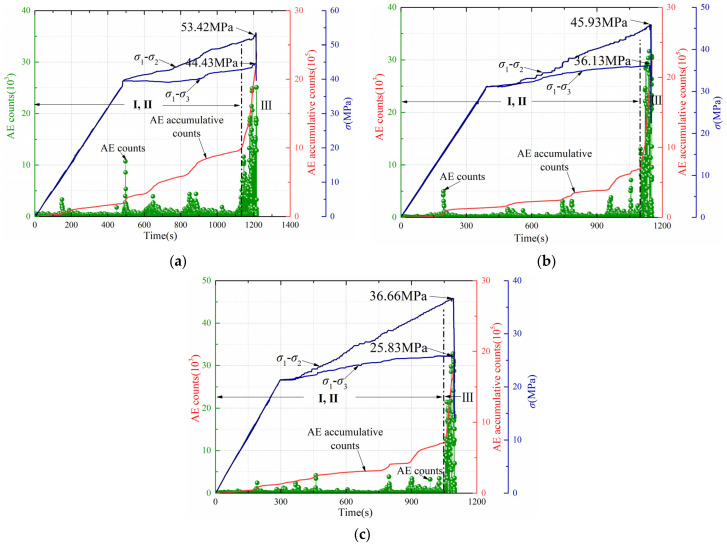
Acoustic emission response during the unloading test. (**a**) Gas pressure of 0.2 MPa. (**b**) Gas pressure of 0.5 MPa. (**c**) Gas pressure of 0.8 MPa.

Figure 8 shows the AE responses during the unloading tests at the different gas pressures. Figure 8a–c correspond to specimens 13, 18, and 19, respectively. Combined with the stress–strain curve of Figure 5, the following rules exist in the unloading tests.

Before the confining stress unloads, the coal is in the linear elastic stage, and the stress–strain curve is linear. At this stage, few paroxysmal AE signals are generated.

The AE signals during the unloading test are slightly different from that during the conventional triaxial test. In conventional triaxial tests, the quiet and active period of AE signals can be easily distinguished, and the cumulative ring-down count curve rises smoothly. However, it is difficult to distinguish between the quiet and active periods in the unloading test because AE ring-down counts increased suddenly and lasted for a long time. The cumulative ring-down count curve shows a sharp upward trend. This increasing trend is more obvious after the unloading begins. After three or four sharp increases, it enters the acceleration period. AE ring-down counts increase sharply, and the cumulative ring-down count curve rises sharply until the stress curve falls. AE ring-down counts gradually decrease after the peak value.

With the increasing of gas pressure, the time of the acceleration period is 111 s, 67 s, and 51 s, respectively. Therefore, the increasing gas pressure will shorten the time until coal rupture, and it will intensify the rate and degree of coal body rupture, so that the failure occurs more rapidly and more intensely.

## 4. Discussion

### 4.1. Damage Based on AE Ring-Down Counts

#### 4.1.1. Damage Model

It is believed that AE signals occur in the process of loading and failure of materials and are proportional to the strain energy released by internal peeling, fracture, and crack propagation [38,39]. Therefore, the number and energy of AE pulses are used to describe the damage characteristics of coal.

According to Kachanov’s theory, the damage variable is defined as *D* [40],

(2) $$D=\frac{A_{d}}{A}$$

where *A_d_* is the area of the microdefect on the load-bearing section and *A* is the area of the initial lossless section.

Assuming that the cumulative AE ring-down count of the complete destruction of the whole cross-sectional area *A* of coal is *N*_0_, then the AE ring-down count *N_m_* of a unit area element failure is [41],

(3) $$N_{m}=\frac{N_{0}}{A}$$

When the damaged section area of coal sample is *A_d_*, the cumulative acoustic emission ring-down count *N_d_* is [42],

(4) $$N_{d}=N_{m}•A_{d}=\frac{N_{0}}{A}A_{d}$$

Consequently, it can be concluded that,

(5) $$\frac{N_{d}}{N_{0}}=\frac{A_{d}}{A}$$

Combining Equations (1) and (5), the damage variable can be expressed as follows:

(6) $$D=\frac{N_{d}}{N_{0}}$$

During the experiments, due to insufficient stiffness of the testing machine or different failure conditions of the coal sample, the sample still has a certain residual strength after the peak, and the damage degree does not reach 1. Therefore, the coal damage can be corrected [43]:

(7) $$D=D_{r}\frac{N_{d}}{N_{0}}$$

where *D_r_* is damage critical value. In the Equation (7), *N*_0_ is the cumulative acoustic emission ring-down count when the damage variable reaches *D_r_*. To simplify the calculation, the damage critical value *D_r_* is calculated as shown Equation (8) [44]. According to Equation (8), when the sample completely loses its bearing capacity, that is, when the residual strength is 0, the damage critical value is 1.

(8) $$D_{r}=1-\frac{\sigma_{r}}{\sigma_{p}}$$

Combining Equations (7) and (8),

(9) $$D=(1-\frac{\sigma_{r}}{\sigma_{p}})\frac{N_{d}}{N_{0}}$$

According to the damage mechanical model [45], the constitutive relationship of coal-rock material is

(10) σ=Eε(1−D)

Combining Equations (9) and (10), the stress can be calculated as follows:

(11) $$\sigma =E\varepsilon [1-(1-\frac{\sigma_{r}}{\sigma_{p}})\frac{N_{d}}{N_{0}}]$$

#### 4.1.2. Damage Analysis

**Figure 9:**
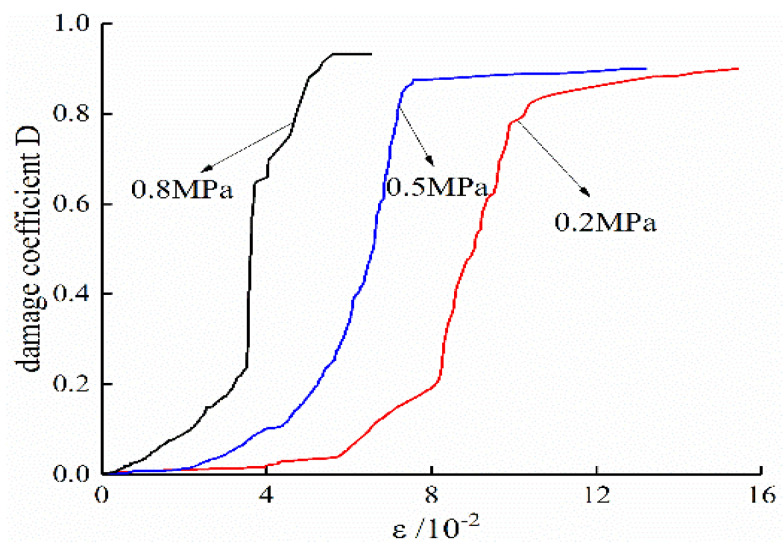
Conventional triaxial test damage coefficient D.

**Figure 10:**
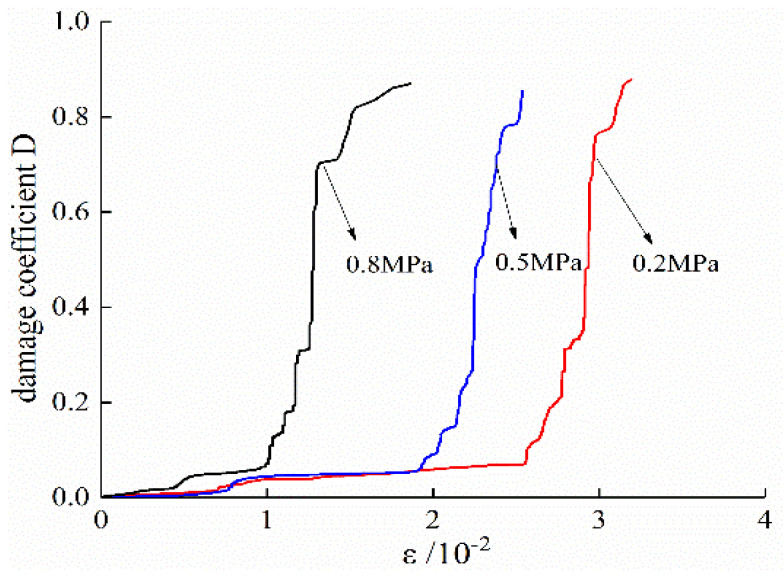
Unloading test damage coefficient D.

**Figure 11:**
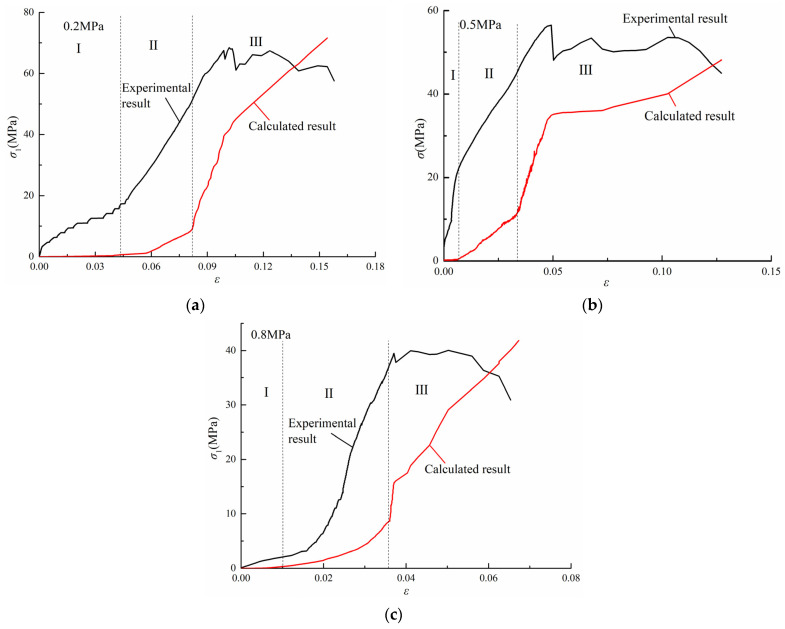
Calculated stress and measured stress for the conventional triaxial test. (**a**) Gas pressure of 0.2 MPa. (**b**) Gas pressure of 0.5 MPa. (**c**) Gas pressure of 0.8 MPa.

**Figure 12:**
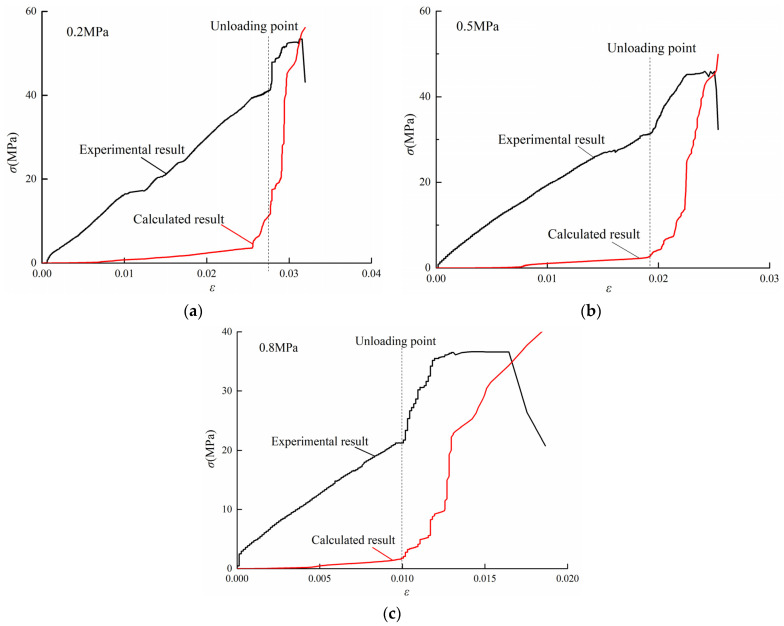
Calculated stress and measured stress for the unloading test. (**a**) Gas pressure of 0.2 MPa. (**b**) Gas pressure of 0.5 MPa. (**c**) Gas pressure of 0.8 MPa.

According to the Formula (3), AE pulses measured on specimens in the conventional triaxial test and unloading tests are normalized. The cumulative damage results of the specimens are shown in Figure 9 and Figure 10. In combination with Formula (4), the strain–damage curve based on the number of AE ring-down counts at different strain levels is obtained and compared with the measured values, as shown in Figure 11 and Figure 12.

In conventional triaxial tests, the coal damage process can be divided into three stages according to the increasing calculated stress values. In the first stage, the coal sample is in the compaction stage and the elastic change stage, and only a small amount of minute damage is generated. In the second stage, internal pore fissures develop, and the damage coefficient *D* increases. In the third stage, which is the precursor of coal failure and instability as mentioned above, the damage coefficient *D* continues to rise rapidly, and the calculated stress also increases rapidly and dramatically. Then, the calculated stress increases more gradually until the measured value reaches a peak.

In the unloading test, most of the specimens before unloading are in the elastic deformation stage. At this stage, only a small amount of damage appears, and the calculated stress increases slowly and slightly. When the confining stress unloaded, the plastic deformation and plateau segment appear. The speed of damage development and the growth rate of calculated stress increase rapidly. After the measured stress reaches its peak value, the calculated stress continues to rise. However, the damage under triaxial conditions is seldom studied. According to the results of this experiment, the residual stress increase in calculated stress after the measured stress reaches its peak may be due to the existence of lateral pressure, which limits the rate of lateral strain. When the coal body breaks macroscopically, there is still a large amount of energy in the coal body that has not yet been released. Moreover, because the sample size was large, there are also more macrofractures in the coal body. After the main fracture occurs, there are still a large number of porous fissures in the process of development and penetration. Therefore, after the measured stress reaches its peak value, the damage does not stop, and the calculated stress will continue to increase. The calculated stress will not decrease until the amount of confining stress relief makes most of the cracks in the coal reach full penetration. However, in this study, the experiment was stopped a few seconds after the measured stress reached its peak value.

### 4.2. Effect of Gas Pressure on Mechanical Behavior of Coal Samples

**Figure 13:**
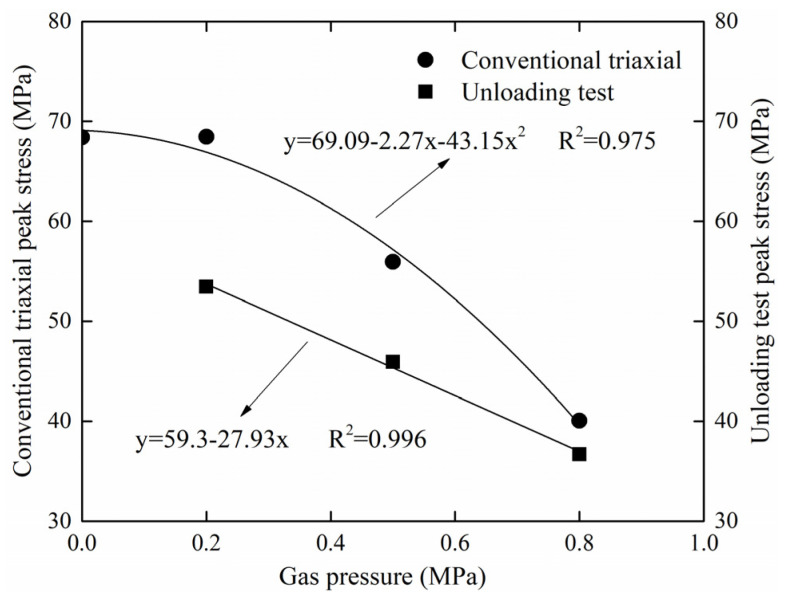
Relationship between peak stress and gas pressure.

In the conventional triaxial test and unloading test, the coal experienced four stages from loading to failure: compaction stage, elastic stage, plastic failure stage, and residual deformation stage. The emphasis of this paper is on the elastic stage and the plastic failure stage. The mechanical model represented by the unloading test is more comparable with the stress state of coal under field mining conditions. Figure 13 is a fitting curve of peak stress and peak stress difference. From the experimental results, the strength of coal decreases with the increasing of gas pressure in both the conventional triaxial test and unloading test. This is consistent with previous studies [46]. The conventional triaxial test fitting curve shows that the strength of coal decreases rapidly as gas pressure increases. The strength of coal in the unloading test is also weakening, but the trend of accelerated decline is subtle.

The decrease in the strength of gas-bearing coal is mainly due to the mechanical properties of the gas in the coal. The gas migration in the coal body occurs mainly by adsorption and seepage. Therefore, during the failure process of gas-bearing coal, gas has two effects on mechanical parameters. First, in the early stage of gas adsorption, the gas entering the interior of the coal body is mainly transported in the free state. The free gas molecules expand the volume of the coal body and reduce the density of the coal body. Moreover, the continuous flow of high-pressure gas accelerates the development of primary and new cracks and accelerates the instability of the coal mass during the process of loading. When the adsorption equilibrium of gas in the coal body has been reached, the surface tension of coal-adsorbed gas and the attraction between coal molecules decrease. Macroscopically, the viscosity between the media particles is reduced, eventually reducing the stress and energy of the coal sample and reducing the peak intensity. These two aspects affect the strength and deformation characteristics of the coal together.

**Figure 14:**
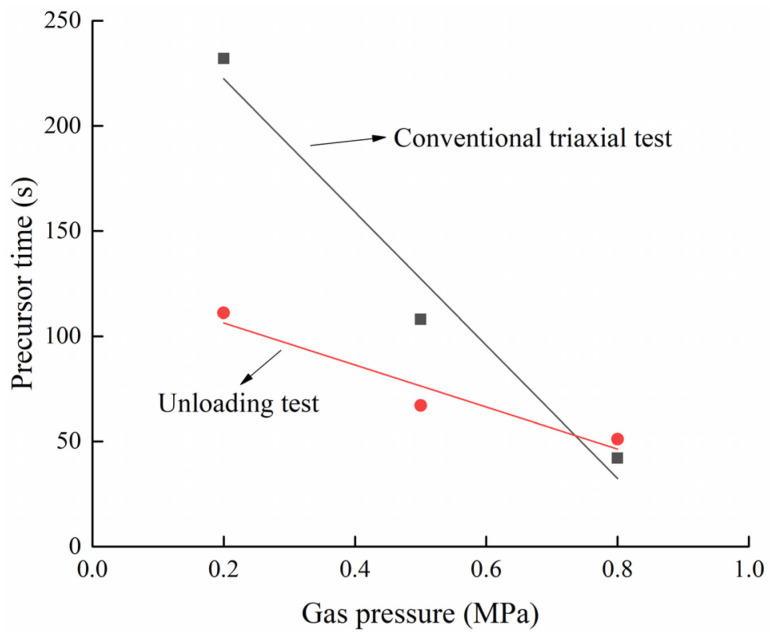
Relationship between precursor signal duration and gas pressure.

Figure 14 is a fitting curve for the duration of precursor signals in the conventional triaxial test and unloading test. Analysis of AE ring-down counts shows that although the final coal samples have been destroyed and destabilized, the precursor signals in the unloading test appear later, and the maintenance time is shorter. The higher gas pressure promotes greater energy release during the process of coal deformation, which amplifies the sudden drop in the stress curve.

## 5. Conclusions

1. Gas pressure obviously influenced the peak strength of gas-bearing coal in the CTS experiments. The peak strength of gas-bearing coal decreases from 68.46 MPa at 0.2 MPa to 40.04 MPa at 0.8 MPa. With the increase in gas pressure, the speed from crack expansion to complete failure of gas-bearing coal is accelerated.
2. Acoustic emission of gas-bearing coal in the CTS experiments can be divided into three stages: quiet period, active period, and acceleration period. The duration time of the quiet period and the active period becomes longer with the increase in gas pressure.
3. The bearing capacity of the samples decreased significantly under the fixed axial unloading confining pressure path. When the gas pressure increases from 0.2 MPa to 0.8 MPa, the maximum of stress difference ($\sigma_{1}$-$\sigma_{2}$) decreases from 53.42 MPa to 36.66 MPa, and the maximum of stress difference ($\sigma_{1}$-$\sigma_{3}$) decreases from 44.43 MPa to 25.83 MPa. Before unloading the confining pressure, the volume of the sample compresses with the increase in axial stress, and the volumetric strain increases continuously. The volume expands when the confining stress unloaded, and the expansion of the sample along the direction of the minimum principal stress is significantly higher than that of the intermediate principal stress. With the increase in gas pressure, the Poisson’s ratio decreases continuously.
4. In the FASUCS experiments, the boundary between the quiet period and the active stage of acoustic emission signals is blurred, and paroxysmal signals are generated occasionally during this process. After entering the rapid fracture stage of the sample, ring-down count and energy of the AE accelerated and increased continuously. With the increase in gas pressure, the duration of the accelerating rising stage is shortened.
5. Taking acoustic emission ring-down count as the damage variable, the stress–strain constitutive equation was established, and the calculated stress variation trend was consistent with the experimental results. The trends in calculated stress and damage coefficient *D* are consistent with the stress path during unloading, and both begin to rise sharply after the sample enters the plastic stage.

## Figures and Tables

**Table 1:** Experimental conditions of coal samples.

Loading Mode	Number	Gas Pressure (MPa)	Confining Stress (MPa)	$\mathrm{Unloading}\;\mathrm{Rate}\;(\sigma_{2}(\mathrm{MPa}/s))$	$\mathrm{Unloading}\;\mathrm{Rate}\;(\sigma_{3}(\mathrm{MPa}/s))$
Conventional triaxial loading	1	0	19	None	None
	2	0			
	3	0			
	4	0.2	19	None	None
	5	0.2			
	6	0.2			
	7	0.5	19	None	None
	8	0.5			
	9	0.5			
	10	0.8	19	None	None
	11	0.8			
	12	0.8			
Fixed axial stress and unloading confining stress	13	0.2	19	0.01	0.005
	14	0.2			
	15	0.2			
	16	0.5	19	0.01	0.005
	17	0.5			
	18	0.5			
	19	0.8	19	0.01	0.005
	20	0.8			
	21	0.8			

## Data Availability

Data are available upon reasonable request.

## References

[1] Liu J., Zhang R., Song D., Wang Z. (2019). Experimental Investigation on Occurrence of Gassy Coal Extrusion in Coalmine. Saf. Sci..

[2] Sa Z., Liu J., Li J., Zhang Y. (2019). Research on Effect of Gas Pressure in the Development Process of Gassy Coal Extrusion. Saf. Sci..

[3] Li X., Cao Z., Xu Y. (2020). Characteristics and Trends of Coal Mine Safety Development. Energy Sources Part A Recovery Util. Environ. Eff..

[4] Liu J., Wang E., Song D., Wang S., Niu Y. (2015). Effect of Rock Strength on Failure Mode and Mechanical Behavior of Composite Samples. Arab. J. Geosci..

[5] Lu S., Zhang Y., Sa Z., Si S., Shu L., Wang L. (2019). Damage-Induced Permeability Model of Coal and Its Application to Gas Predrainage in Combination of Soft Coal and Hard Coal. Energy Sci. Eng..

[6] Lu S., Wang C., Liu Q., Zhang Y., Liu J., Sa Z., Wang L. (2019). Numerical Assessment of the Energy Instability of Gas Outburst of Deformed and Normal Coal Combinations during Mining. Process Saf. Environ. Protect..

[7] Lu S., Zhang Y., Sa Z., Si S. (2019). Evaluation of the Effect of Adsorbed Gas and Free Gas on Mechanical Properties of Coal. Environ. Earth Sci..

[8] Xie H.-P., Zhou H.-W., Liu J.-F., Gao F., Zhang R., Xue D.-J., Zhang Y. (2011). Mining-Induced Mechanical Behavior in Coal Seams under Different Mining Layouts. Meitan Xuebao J. China Coal Soc..

[9] Wen Z., Xing E., Shi S., Jiang Y. (2019). Overlying Strata Structural Modeling and Support Applicability Analysis for Large Mining-Height Stopes. J. Loss Prev. Process Ind..

[10] Zhang M., Lin M., Zhu H., Zhou D., Wang L. (2018). An Experimental Study of the Damage Characteristics of Gas-Containing Coal under the Conditions of Different Loading and Unloading Rates. J. Loss Prev. Process Ind..

[11] Lu S., Wang C., Li M., Sa Z., Zhang Y., Liu J., Wang H., Wang S. (2021). Gas Time-Dependent Diffusion in Pores of Deformed Coal Particles: Model Development and Analysis. Fuel.

[12] Qiu L., Song D., He X., Wan E., Li Z., Yi S., We M., Li Y. (2020). Multifractal of Electromagnetic Waveform and Spectrum About Coal Rock Samples Subjected to Uniaxial Compression. Fractals-Complex Geom. Patterns Scaling Nat. Soc..

[13] Qiu L., Liu Z., Wang E., He X., Feng J., Li B. (2020). Early-Warning of Rock Burst in Coal Mine by Low-Frequency Electromagnetic Radiation. Eng. Geol..

[14] Kwaśniewski M. (2013). Recent Advances in Studies of the Strength of Rocks Under True Triaxial Compression Conditions / POSTĘPY W Badaniach Nad WYTRZYMAŁOŚCIĄ SKAŁ W Warunkach Prawdziwego TRÓJOSIOWEGO ŚCISKANIA. Arch. Min. Sci..

[15] Taheri A., Squires J., Meng Z., Zhang Z. (2017). Mechanical Properties of Brown Coal under Different Loading Conditions. Int. J. Geomech..

[16] Palm J.H. (1951). Stress-Strain Relations for Uniform Monotonic Deformation under Triaxial Loading. Appl. Sci. Res..

[17] Taheri A., Sasaki Y., Tatsuoka F., Watanabe K. (2012). Strength and Deformation Characteristics of Cement-Mixed Gravelly Soil in Multiple-Step Triaxial Compression. Soils Found..

[18] Bruning T., Karakus M., Nguyen G.D., Goodchild D. (2018). Experimental Study on the Damage Evolution of Brittle Rock Under Triaxial Confinement with Full Circumferential Strain Control. Rock Mech. Rock Eng..

[19] Chen J., Du C., Jiang D., Fan J., He Y. (2016). The Mechanical Properties of Rock Salt under Cyclic Loading-Unloading Experiments. Geomech. Eng..

[20] Pirzada M.A., Zoorabadi M., Ramandi H.L., Canbulat I., Roshan H. (2018). CO2 Sorption Induced Damage in Coals in Unconfined and Confined Stress States: A Micrometer to Core Scale Investigation. Int. J. Coal Geol..

[21] Wang W.-Z., Yin G.-Z., Wang D.-K., Qin H. (2010). A Visco-Elasto-Plastic Creep Model of Outburst Prone Coal under Triaxial Compression. Chongqing Daxue Xuebao J. Chongqing Univ..

[22] Xu Y., Kang H., Zhang H., Wang W. (2014). Experimental Study of Mechanical Properties of Coal Containing Gas under Unloading Conditions. Yanshilixue Yu Gongcheng Xuebao Chin. J. Rock Mech. Eng..

[23] He S., Song D., Li Z., He X., Chen J., Li D., Tian X. (2019). Precursor of Spatio-Temporal Evolution Law of MS and AE Activities for Rock Burst Warning in Steeply Inclined and Extremely Thick Coal Seams Under Caving Mining Conditions. Rock Mech. Rock Eng..

[24] Wang X., Wen Z., Jiang Y., Huang H. (2018). Experimental Study on Mechanical and Acoustic Emission Characteristics of Rock-Like Material Under Non-Uniformly Distributed Loads. Rock Mech. Rock Eng..

[25] Li X., Chen S., Wang E., Li Z. (2021). Rockburst Mechanism in Coal Rock with Structural Surface and the Microseismic (MS) and Electromagnetic Radiation (EMR) Response. Eng. Fail. Anal..

[26] Dunegan H.L., Harris D.O., Tatro C.A. (1968). Fracture Analysis by Use of Acoustic Emission. Eng. Fract. Mech..

[27] Kong X., Wang E., He X., Liu X., Li D., Liu Q. (2018). Cracks Evolution and Multifractal of Acoustic Emission Energy during Coal Loading. Geomech. Eng..

[28] Jin P., Wang E., Song D. (2017). Study on Correlation of Acoustic Emission and Plastic Strain Based on Coal-Rock Damage Theory. Geomech. Eng..

[29] Wang X., Liu X., Wang E., Li X., Zhang X., Zhang C., Kong B. (2017). Experimental Research of the AE Responses and Fracture Evolution Characteristics for Sand-Paraffin Similar Material. Constr. Build. Mater..

[30] Bismayer U. (2017). Early Warning Signs for Mining Accidents: Detecting Crackling Noise. Am. Mineral..

[31] Yang H., Wen G., Hu Q., Li Y., Dai L. (2018). Experimental Investigation on Influence Factors of Acoustic Emission Activity in Coal Failure Process. Energies.

[32] Kurita K., Fujii N. (1979). Stress Memory of Crystalline Rocks in Acoustic Emission. Geophys. Res. Lett..

[33] Shkuratnik V.L., Filimonov Y.L., Kuchurin S.V. (2004). Experimental Investigations into Acoustic Emission in Coal Samples under Uniaxial Loading. J. Min. Sci..

[34] Kong X., Wang E., Li S., Lin H., Xiao P., Zhang K. (2019). Fractals and Chaos Characteristics of Acoustic Emission Energy About Gas-Bearing Coal During Loaded Failure. Fractals-Complex Geom. Patterns Scaling Nat. Soc..

[35] Su C., Gao B., Nan H., Li X. (2009). Experimental Study on Acoustic Emission Characteristics during Deformation and Failure Processes of Coal Samples under Different Stress Paths. Yanshilixue Yu Gongcheng Xuebao Chin. J. Rock Mech. Eng..

[36] Browning J., Meredith P.G., Stuart C.E., Healy D., Harland S., Mitchell T.M. (2017). Acoustic Characterization of Crack Damage Evolution in Sandstone Deformed under Conventional and True Triaxial Loading. J. Geophys. Res. Solid Earth.

[37] Gao C.-Y., Xu J., He P., Liu J.-F. (2005). Study on Mechanical Properties of Marble under Loading and Unloading Conditions. Yanshilixue Yu Gongcheng Xuebao Chin. J. Rock Mech. Eng..

[38] Zhang R., Liu J., Sa Z., Wang Z., Lu S., Wang C. (2020). Experimental Investigation on Multi-Fractal Characteristics of Acoustic Emission of Coal Samples Subjected to True Triaxial Loading-Unloading. Fractals-Complex Geom. Patterns Scaling Nat. Soc..

[39] Zhang R., Liu J., Sa Z., Wang Z., Lu S., Lv Z. (2021). Fractal Characteristics of Acoustic Emission of Gas-Bearing Coal Subjected to True Triaxial Loading. Measurement.

[40] Kachanov M. (1992). Effective Elastic Properties of Cracked Solids: Critical Review of Some Basic Concepts. Appl. Mech. Rev..

[41] Yamada I., Masuda K., Mizutani H. (1989). Electromagnetic and Acoustic Emission Associated with Rock Fracture. Phys. Earth Planet. Inter..

[42] Wang E., He X., Wei J., Nie B., Song D. (2011). Electromagnetic Emission Graded Warning Model and Its Applications against Coal Rock Dynamic Collapses. Int. J. Rock Mech. Min. Sci..

[43] Liu B., Huang J., Wang Z., Liu L. (2009). Study on Damage Evolution and Acoustic Emission Character of Coal-Rock under Uniaxial Compression. Yanshilixue Yu Gongcheng Xuebao Chin. J. Rock Mech. Eng..

[44] Niu Y., Wang C., Wang E., Li Z. (2019). Experimental Study on the Damage Evolution of Gas-Bearing Coal and Its Electric Potential Response. Rock Mech. Rock Eng..

[45] Krajcinovic D., Silva M.A.G. (1982). Statistical Aspects of the Continuous Damage Theory. Int. J. Solids Struct..

[46] Meng L., Wang H.-W., Li X.-H., Zhao Y.-X. (2014). Investigation on Acoustic Emission Characteristics in Failure Process of Coal Absorbed Methane. Meitan Xuebao J. China Coal Soc..

